# Complete chloroplast genome of *Rhododendron pulchrum*, an ornamental medicinal and food tree

**DOI:** 10.1080/23802359.2019.1676181

**Published:** 2019-10-11

**Authors:** Jian Shuang Shen, Xue Qin Li, Xiang Tao Zhu, Xiao Ling Huang, Song Heng Jin

**Affiliations:** a Jiyang College, Zhejiang A&F University, Zhuji, China;; b The Nurturing Station for the State Key Laboratory of Subtropical Silviculture, School of Forestry and Biotechnology, Zhejiang A&F University, Lin’an, China

**Keywords:** *Rhododendron pulchrum*, chloroplast genome, phylogenetic analysis

## Abstract

The semi-evergreen azalea, *Rhododendron pulchrum*, a valuable horticultural and medicinal plant species. Using next-generation sequencing, applying a combination of de novo and reference-guided assembly, we sequenced its complete chloroplast genome. Our study reveals that *R. pulchrum* have a typical cp genome of 136,249 bp in length, without inverted repeat regions. A total of 73 genes, 42 of which are protein coding genes, 29 tRNA genes, two rRNA genes were identified. The GC content of the whole genome is 35.98%. Phylogenetic analysis indicates that *R. pulchrum* is closely related to the species of *Vaccinium oldhamii* and *Vaccinium macrocarpon*.

*Rhododendron pulchrum*, as the important horticultural, medicinal, and food plants, belongs to the genus *Ericaceae*, were widely distributed in temperate regions of Europe, Asia and North America (Galle 1985). Previous studies have focussed on its biology and physiology (Zhang 2012). However, there has no study on its chloroplast (cp) genome. Through chloroplast genome could provide valuable genetic sequence data for the studies on species identification, phylogeny, and biological (Zhu 2018), owing to its conserved genome structures and comparatively high substitution rates (Wu and Ge 2012; Qin et al. 2018). Herein, we report the complete chloroplast genome of *R. pulchrum*. The genetic sequence information will have valuable applications in phylogenetic evolution, molecular markers and chloroplast genetic engineering studies of *Rhododendron.*

The fresh leaves of *R. pulchrum* were sampled from Jiyang College of Zhejiang A&F University (stored in *Rhododendron* Germplasm Resource nursery in Hangzhou Botanical Garden Mem and the specimen Accession number is HZ041286, N30°15′8.65″, E120°7′11.09″) and were used for the total genomic DNA extraction with the modified method CTAB (Doyle 1987). The whole-genome sequencing was conducted with 250 bp pair-end reads on the Illumina Hiseq 2500 Platform (Nanjing, China), yielding at least 9.82 GB clean data, which was used for the cp genome de novo assembly using the programme NOVOPlasty (Dierckxsens et al. 2017). CpGAVAS, DOGMA (http://dogma.ccbb.utexas.edu/) and BLAST were used to annotate the sequences (Chang et al. 2012; Wyman et al. 2004). The mVISTA (Mayor et al. 2000) programme was applied to compare the complete cp genome of *R. pulchrum* to the other 13 reported chloroplast genomes of its related species.

The cp genome of *R. pulchrum* was 136,249 bp in length (the accession number MN182619), which did not contain inverted repeats. The cpDNA contains 73 genes, comprising 42 protein-coding genes, 29 tRNA genes, two rRNA genes. Among the annotated genes, *trn*V-UAC, *trn*L-UAA, *ndh*A, *trn*A-UGC, *trn*I-GAU, and *trn*G-UCC contain one intron, and *ycf*3 contain two introns. The overall GC content of the plastome is 35.98%. Further, the phylogenetic analysis of 14 plastid genomes showed that the cp genome of *R. pulchrum* is closely related to the species of *Vaccinium oldhamii* and *Vaccinium macrocarpon* (Figure 1).

**Figure 1.**
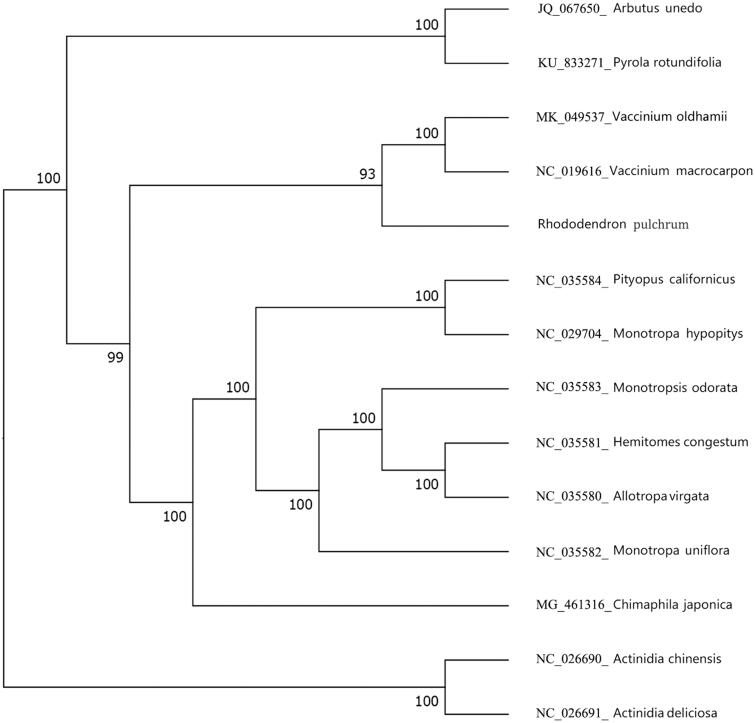
Phylogenetic tree based on 14 complete chloroplast genome sequences.

The *R. pulchrum* cp genome information will also provide fundamental data for the conservation and utilisation and for population genomic and phylogenomic studies of *R. pulchrum*.
